# Comparison of Fourier-Domain and Time-Domain Optical Coherence Tomography in the Measurement of Thinnest Corneal Thickness in Keratoconus

**DOI:** 10.1155/2015/402925

**Published:** 2015-06-25

**Authors:** Chunxiao Wang, Xueying Xia, Bishan Tian, Shiyou Zhou

**Affiliations:** ^1^State Key Laboratory of Ophthalmology, Zhongshan Ophthalmic Center, Sun Yat-sen University, Guangzhou, Guangdong 510060, China; ^2^Guangdong Women and Children Hospital, Guangzhou, Guangdong 510000, China

## Abstract

*Objective*. To compare fourier-domain optical coherence tomography (FD-OCT) and time-domain OCT (TD-OCT) in the determination of thinnest corneal thickness (TCT). *Methods*. This study included 55 keratoconus patients and 50 healthy volunteers. The RTVue-OCT (FD-OCT) and Visante-OCT (TD-OCT) were used for the measurement of the TCT. Three consecutive scans were performed. The comparison and agreement between the two modalities were analyzed by paired *t*-test, the Pearson correlation, intraclass correlation coefficient (ICC), and Bland-Altman plots. intraobserver repeatability was analyzed by the intraobserver within-subject standard deviation (*S*_(*w*)_), coefficient of variation (CV_(*w*)_), and ICC. *Results*. The TCT value of normal corneas was higher by RTVue-OCT (530.4 ± 19.7 *μ*m) than Visante-OCT (521.5 ± 18.3 *μ*m) (*p* < 0.001). For keratoconus eyes, the TCT was 425.0 ± 58.2 *μ*m and 424.4 ± 55.7 *μ*m (difference being 0.6 ± 10.2 *μ*m, *p* = 0.604). Strong correlation (*r* = 0.938∼0.985) (ICC = 0.915–0.984) was observed between the two OCTs, and each OCT exhibited excellent repeatability in determining the TCT in all subjects (ICC = 0.984–0.994). *Conclusions*. The values of TCT obtained from RTVue-OCT and Visante-OCT were highly correlated; however, the two values were different. Both OCT instruments exhibited good intraobserver reliability. The existence of systematic differences suggested that the two instruments cannot be used interchangeably.

## 1. Introduction

Corneal thinning is one of the key pathological features in various corneal degenerative diseases, such as keratoconus [1]. Evaluation of corneal thickness is essential in the diagnosis of keratoconus. The thinnest corneal thickness (TCT) measurement has emerged as an efficient diagnostic parameter in cases where the classical topographic keratoconus index is not suitable [2]. In addition, measurement of TCT also provides a systematic approach for designing surgical interventions in keratoconus, such as deep lamellar keratoplasty, collagen cross-linking, and intrastromal ring placement [3, 4].

Until recently, the thinnest region of cornea was considered as the center of the cornea. However, with the advancements in the anterior segment imaging techniques, it was recognized that a point inferior-temporal to the corneal center was the thinnest region of the cornea [5, 6]. Currently, different imaging instruments are used in the measurement of TCT, including placido-disc technology (Orbscan II), rotating Scheimpflug camera technology (Pentacam), and optical coherence tomography (OCT) technology.

The OCT technology is superior to the other two imaging instruments for accurately mapping corneal thickness of the corneas with opacities or interface anomalies [7–9]. The OCT technique generates cross-sectional images using low-coherence interferometry and by comparing the optical backscattered light from the tissue structures with reference lights [10, 11]. It has two classifications: fourier-domain OCT (FD-OCT) and time-domain OCT (TD-OCT). FD-OCT is associated with rapid scan speed and higher resolution as compared to TD-OCT. The scan speed of TD-OCT systems depends on the mechanical cycle time of the moving reference mirror driver, whereas in FD-OCT, the reference mirror is fixed, which assists in sampling multiple points from the ocular structures simultaneously. Thus, relatively high acquisition speed (up to 26,000 A-scans per second), which is ~100 times faster than TD-OCT, is obtained with FD-OCT. The resulting high scan speed with FD-OCT distinctly improves resolution, significantly reduces motion artifacts, and increases signal-to-noise ratio [12, 13]. By contrast, TD-OCT can penetrate deeper into the sclera, iris, and cornea than FD-OCT, owing to longer wavelength of its detector [10, 14]. Although there were various studies that compared the two imaging techniques in posterior segment of the eye, the determination of TCT was rarely studied. In this study, we compared and evaluated FD-OCT and TD-OCT in the determination of TCT for keratoconic and normal eyes. The intraobserver repeatability of each of the OCTs was also measured to demonstrate the reliability of each method.

## 2. Materials and Methods

This prospective, cross-sectional study included 55 keratoconus patients (82 eyes) and 50 volunteers (50 eyes). The keratoconus patients were consulted to Zhongshan Ophthalmic Center from January 2012 to July 2013, and the volunteers were normal people who consulted the institute for laser in situ keratomileusis (LASIK) treatment.

For specific comparison, eyes of the patients with keratoconus were classified according to the Amsler-Krumeich classification into two categories based on the TCT [15]. Subgroup 1 included patients with stage I and stage II of keratoconus (TCT above 400 *μ*m), whereas in subgroup 2, patients with stage III and IV of keratoconus (TCT below 400 *μ*m) were included. Informed consent was obtained from each of the participants. This study followed the guidelines of the Declaration of Helsinki and was approved by the Institutional Review Board of Zhongshan Ophthalmic Center, China.

Each of the participants underwent comprehensive ophthalmic examination, which included visual acuity, intraocular pressure, slit lamp biomicroscopy, and topographic examination. Inclusion criteria for keratoconus patients included abnormal topographic appearance with at least one clinical sign upon slit-lamp evaluation, such as Vogt striae, Fleischer's ring, Munson's sign, Rizutti's sign, apical thinning, and mild apical scarring. However, patients with keratoconic eyes, characterized by acute hydrops, previous surgical treatments, and history of corneal disease, were excluded from the study. None of the volunteers had ocular disorders, except for refractive error. The duration of examinations was three hours (from 9:00 AM to 12:00 PM), to minimize the effect of diurnal variation on the corneal thickness. In each of the participants, FD-OCT was performed after a rest of 15 minutes, which was followed by TD-OCT. Three consecutive scans were performed on each of the participants to assess intraobserver repeatability. The average of data thus obtained was used for comparison.

### 2.1. Fourier-Domain OCT

**Figure 1:**
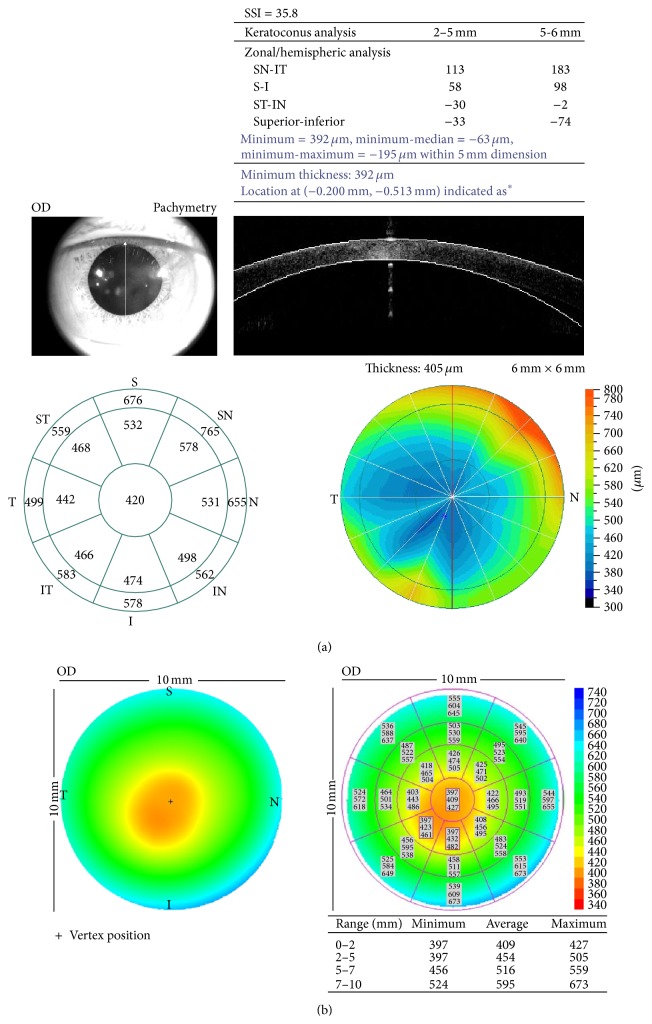
Corneal pachymetry of a patient with keratoconus generated by the (a) RTVue-OCT and (b) Visante-OCT. (a) Minimum of corneal thickness can be acquired from the table of “keratoconus analysis” on the upper right and the thinnest point is also marked on the pachymetric map on the lower right. A real-time monitoring on the upper left is used for pupil centering. (b) Minimum of corneal thickness in different regions is demonstrated on the table on the lower right and the thinnest point is also marked on the pachymetric map on the left.

The RTVue-OCT (Model RT100, Optovue Inc, Fremont, CA, USA) system was used for measuring TCT. The scan beam wavelength, scan speed, axial resolution, and transverse resolution were 840 nm, 26,000 A-scans per second, 5 *μ*m, and 8 *μ*m, respectively. The RTVue-OCT was originally designed for retinal imaging. An additional lens adapter, known as low-magnification cornea anterior module (CAM-L), was used for anterior segment imaging. Using the corneal pachymetry protocol, the “thinnest” values were automatically generated in the “keratoconus analysis” table (Figure 1(a)). For RTVue-OCT examination, the subject was kept in sitting position with an external fixation, without the application of topical anesthesia, and three consecutive scans were carried out at an interval of 4-5 seconds.

### 2.2. Time-Domain OCT

The Visante-OCT (Carl Zeiss Meditec Inc, Dublin, CA, USA) system was used for measuring TCT. The wavelength and scan speed of the Visante-OCT were 1310 nm and 2048 A-scans per second, respectively. For assessment, the global corneal pachymetry protocol was used. There were 8 radial scans centered on the cornea. Each scan line was 10 mm long, with a transverse resolution of 60 *μ*m and a vertical resolution of 18 *μ*m. For Visante-OCT examination, the participants were asked to sit with a headrest and the internal fixation was used. Three consecutive scans were carried out at an interval of 4-5 seconds. The output table displayed global corneal pachymetry. Maximum, average, and thinnest readings of corneal thickness in four concentric circles (0–2, 2–5, 5–7, and 7–10 mm) were computed automatically. The least reading in the table was chosen for analysis (Figure 1(b)).

### 2.3. Statistical Analysis

Statistical analysis was performed using SPSS software for Microsoft Windows (version 18.0, SPSS Inc., Chicago, IL, USA). The Kolmogorov-Smirnov test was used for normality testing of the data. Paired *t*-tests were used to analyze the difference between the data obtained from RTVue-OCT and Visante-OCT. The Pearson correlation coefficient and intraclass correlation coefficient (ICC) were calculated to assess the relationship between the data from the two instruments. Bland-Altman plots and 95% limits of agreement (LoA) were used to demonstrate the agreement between the data. The within-subjects SD, CV_(*w*)_, and ICC were calculated to determine the intraobserver repeatability. A *p* value of ≤0.05 was considered as statistically significant.

## 3. Results

### 3.1. Demography

Eighty-two eyes from 55 patients with keratoconus (40 males and 15 females) and 50 normal eyes from 50 volunteers (35 males and 20 females) were compared in this study. The average age of patients and volunteers was 24.6 ± 7.5 years and 23.2 ± 6.8 years, respectively. The mean of the maximum simulated keratometric reading for keratoconic eyes and normal eyes was 55.40 ± 5.7 D and 44.2 ± 0.8 D, respectively.

### 3.2. Measurement of Thinnest Corneal Thickness

For normal eyes, TCT obtained from RTVue-OCT was significantly higher than Visante-OCT TCT (difference = 8.9 ± 6.9 *μ*m; *p* < 0.001; paired *t*-test). For keratoconic eyes, the difference between the TCTs decreased from 8.9 ± 6.9 *μ*m to 0.6 ± 10.2 *μ*m, with no statistical significance (*p* = 0.604; paired *t*-test). In keratoconic group, subgroup 1 included 45 eyes, whereas subgroup 2 included 37 eyes. In subgroup 1, higher TCT was obtained from RTVue-OCT than Visante-OCT, (difference = 3.2 ± 7.7 *μ*m; *p* = 0.009; paired *t*-test), whereas in subgroup 2, the lower TCT was obtained with RTVue-OCT (difference = −2.3 ± 11.8 *μ*m; *p* = 0.223; paired *t*-test) (Table 1). For both OCTs, the TCT of keratoconic eyes was significantly less than that of normal eyes (*p* < 0.001, independent *t*-test).

**Figure 2:**
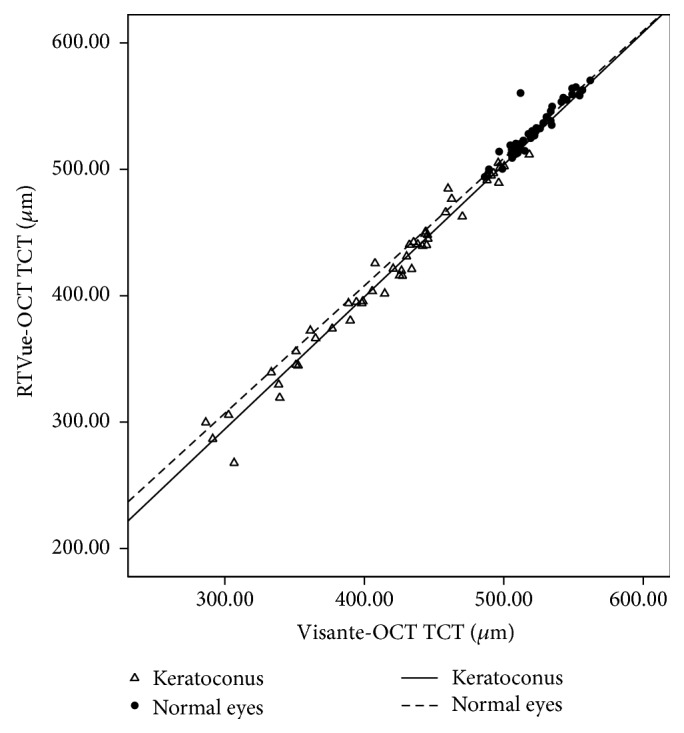
Scatterplot showing the relationship of the TCT values between RTVue-OCT and Visante-OCT in keratoconic and normal eyes.

**Figure 3:**
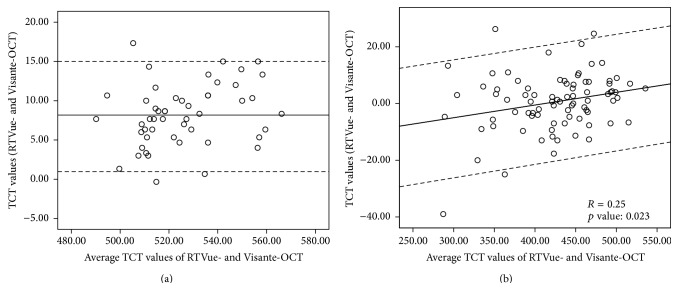
Bland-Altman plots showing the agreement between the RTVue-OCT and Visante-OCT measurements of the TCT. (a) Normal eyes. (b) Keratoconic eyes.

The two OCT systems exhibited strong correlation in the measurement of TCT for both normal and keratoconic eyes (Figure 2; correlation coefficient (*r*) = 0.938–0.985) (ICC = 0.915–0.984) (Table 1). Bland-Altman plot was used to demonstrate the effect of average TCT on the difference between the two methods (Figure 3). A constant bias (mean difference = 8.20 *μ*m) was observed for normal eyes, as consistently higher TCT values were obtained by RTVue-OCT as compared to Visante-OCT (95% LoA width = 13.97 *μ*m; range 1.03–15.0 *μ*m) (Figure 3(a)). For keratoconic eyes, a proportional bias was observed between the RTVue-OCT and Visante-OCT (*R* = 0.25, *p* = 0.023; 95% LoA width = 34.42 *μ*m; range −16.97–17.45 *μ*m) (Figure 3(b)), with a mean difference of 0.59 ± 10.19 *μ*m.

### 3.3. Assessment of Intraobserver Repeatability

Both OCTs exhibited good intraobserver repeatability in measuring TCT. For normal eyes, the mean difference of repeat measurements was 1.2 ± 0.8 *μ*m with RTVue-OCT and 1.8 ± 1.0 *μ*m with Visante-OCT, whereas for keratoconic eyes, the mean difference was 5.2 ± 5.6 *μ*m and 4.6 ± 4.5 *μ*m, respectively. For normal eyes, the intraobserver repeatability was better with RTVue-OCT, whereas in keratoconic eyes, Visante-OCT demonstrated better intraobserver repeatability (Table 2).

## 4. Discussion

The assessment of TCT is essential for early diagnosis and surgical planning for patients with corneal thinning diseases, such as keratoconus [2–4]. The onset of OCT technique has provided clinicians an ideal method to measure TCT for both normal corneas and corneas with slight stromal opacities [7, 8]. Our study reported the comparison between TCTs obtained from FD-OCT and TD-OCT systems in keratoconus. The results have demonstrated good correlation between the two OCT systems and intraobserver repeatability of each of the OCTs. However, the existence of systemic biases indicated that the two imaging techniques cannot be used interchangeably.

Various studies have compared the application of FD-OCT and TD-OCT in posterior segment of eyes [16–18]; however, only few studies have considered the anterior segment of eyes. In this study, we observed significantly higher readings of TCT from RTVue-OCT than Visante-OCT for normal eyes (mean difference = 8.9 ± 6.9 *μ*m). The results were consistent with other findings on corneal thickness measurement for normal eyes. Prakash et al. reported mean differences of TCT and central corneal thickness (CCT) as obtained from RTVue-OCT and Visante-OCT. The results demonstrated a significant mean difference of 19.3 ± 24 *μ*m and 5.88 ± 9.1 *μ*m, respectively [19]. Huang et al. also reported the mean CCT difference of 1.13 ± 5.53 *μ*m between the two modalities [20]. The possible explanation for this disparity can be attributed to different principles of imaging and segmentation algorithms. Li et al. demonstrated that the automated algorithm of Visante-OCT delineated the anterior corneal boundary slightly below the anterior corneal surface, thus affecting the corneal thickness values [21]. Fourier-domain optical coherence tomography was associated with higher resolution as compared to Visante-OCT and provided an increased signal-to-noise ratio that provided enhanced edge definition of the corneal surfaces [10, 12, 13].

The present study determined the corneal thickness and abnormal stromal structures in patients with keratoconus. Noticeably, the discrepancy between RTVue-OCT and Visante-OCT decreased when the relative thinner corneas were measured. The proportional bias between the two techniques, indicating the difference in measuring TCT between RTVue-OCT and Visante-OCT, is illustrated in Figure 2. We postulated that the difference was attributed to abnormal OCT hyperreflectivity of thinner regions in keratoconic corneas. In keratoconic eyes, corneal structures, including Bowman's membrane, stromal layers, and Descemet's membrane, underwent pathological changes with corneal thinning [22], which affected the refractive index (RI) of the corneas, thereby altering thickness of the OCT. Corneal thickness obtained from OCT technique is based on the RI of the interface. For normal corneas, the RI of corneal stromal layers is assumed to be constant. However, for keratoconic eyes, posterior displacement of Bowman's membrane or stromal scarring leads to abnormal reflectivity in OCT images [23]. The heterogeneous stroma may cause fluctuations in the RI and interferes with the measurement of corneal thickness by OCT.

From the best-fit lines for TCT values in Figure 1, we recognized that the correlation between the two techniques was good for all the participants (*r* = 0.938–0.985), thereby suggesting a consistent and narrowly distributed set of values. Although data from the two OCT systems cannot be used interchangeably, the equations for TCT may be applicable in converting data acquired from one modality to the other. Contrary to our results, Prakash et al. reported that the correlation, however, was less pronounced in the assessment of normal eyes (*R* ^2^ = 0.58) [19]. Based on these findings, we opined that the difference in the races and testing standards might attribute to the resulting difference. Despite using the similar OCTs, the resulting TCT values were different. In this study, the TCT values as obtained from RTVue-OCT and Visante-OCT were 530.4 ± 19.7 *μ*m and 521.5 ± 18.3 *μ*m, respectively, whereas the values were 512.4 ± 34.7 *μ*m and 493.1 ± 34.8 *μ*m in the study conducted by Prakash et al.

The intraobserver repeatability for the OCT systems was found to be good (Table 2). However, the repeatability of each of the OCTs for keratoconic eyes was not as good as that for normal eyes. The significant difference in corneal thickness was attributed to the significant variations in central 5 mm region in the corneal contour of the keratoconic eyes [24].

For normal eyes, the data obtained from RTVue-OCT was found to be more reliable than Visante-OCT data, owing to the rapid scan speed and higher resolution in the detection of corneal edge [25]. On the other hand, for keratoconic eyes, the repeatability of RTVue-OCT was less pronounced. Thus, we concluded that RTVue-OCT was more susceptible to corneal scarring in the determination of corneal thickness. Although the FD-OCT was associated with higher clinical utility in mild keratoconus evaluation and normal eyes, it was found to be unsuitable in keratoconus with corneal scarring.

In this study, gold standard method was not included in the determination of TCT. In addition, it was not established whether the values obtained by RTVue-OCT or Visante-OCT were accurate, especially in different conditions (normal and keratoconus eyes). Another limitation is that the keratoconic eyes were not classified according to the corneal interface anomalies. This might have affected the correlation between the data from the OCT systems. Nevertheless, the results did shed light on the comparison between FD-OCT and TD-OCT in the measurement of TCT. In addition, our results have demonstrated that the TCT values obtained from both the methods are not exchangeable.

The present study demonstrated good correlation between the TCT values obtained from RTVue-OCT and Visante-OCT. The OCTs exhibited good intraobserver repeatability; however, small systematic differences were observed between the two OCT systems. Moreover, data from the two instruments cannot be used interchangeably, or in case of interchangeability the measured data should be corrected by the offset.

## Figures and Tables

**Table 1:** Difference and correlation analysis of the TCT from the RTVue-OCT and Visante-OCT.

	RTVue-OCT				Visante-OCT				Difference			Correlation		ICC
	Mean	SD	95% CI-U	95% CI-L	Mean	SD	95% CI-U	95% CI-L	Mean	SD	*p*	*r*	*p*	(95% LoA)
Normal eyes	530.4	19.7	500.2	564.5	521.5	18.3	493.4	555.2	8.9	6.9	<0.001	0.938	<0.001	0.915 (0.906–0.952)
Keratoconus	425.0	58.2	307.7	504.6	424.4	55.7	310.7	499.8	0.6	10.2	0.604	0.985	<0.001	0.984 (0.976–0.990)
Subgroup 1 (keratoconus)	469.5	26.5	433.4	518.6	466.2	25.9	430.7	517.3	3.2	7.7	0.009	0.957	<0.001	0.950 (0.899–0.974)
Subgroup 2 (keratoconus)	376.0	41.7	286.7	425.7	378.3	41.3	291.3	423.0	−2.3	11.8	0.223	0.960	<0.001	0.959 (0.924–0.978)

**Table 2:** Analysis of intraobserver repeatability of each OCT for normal and keratoconic eyes.

	Normal eyes		Keratoconus	
	RTVue-OCT	Visante-OCT	RTVue-OCT	Visante-OCT
*S*_(*w*)_	1.23	1.76	5.17	4.65
CV_(*w*)_	0.2%	0.3%	1.2%	1.1%
ICC (95% LoA)	0.994 (0.991–0.997)	0.989 (0.983–0.993)	0.984 (0.976–0.989)	0.987 (0.981–0.991)
